# Effects of the Computer Desk Level on the Musculoskeletal Discomfort of Neck and Upper Extremities and EMG Activities in Patients with Spinal Cord Injuries

**DOI:** 10.1155/2019/3026150

**Published:** 2019-02-03

**Authors:** Bo-Ra Kang, Jin-Gang Her, Ju-Sang Lee, Tae-Sung Ko, Young-Youl You

**Affiliations:** ^1^Department of Occupational Therapy, Yonseimadu Hospital, Goyang, Republic of Korea; ^2^Department of Rehabilitation Therapy, Graduate School of Hallym University, Chuncheon, Republic of Korea; ^3^Department of Physical Therapy, Hallym Polytechnic University, Chuncheon, Republic of Korea; ^4^Department of Physical Therapy, Daewon University College, Jecheon, Republic of Korea; ^5^Department of Physical Therapy, Bronco Memorial Hospital, Hwaseong, Republic of Korea

## Abstract

**Background:**

Computers are used as a means of social communication, for work and other purposes. However, patients with spinal cord injuries may have a higher risk than normal individuals with musculoskeletal problems when using computers owing to their inability to control respective postures due to problems in motor and sensory functioning.

**Objectives:**

This study is aimed at identifying the effect of computer desk heights on musculoskeletal discomforts of the neck and upper extremities and EMG activities in patients with spinal cord (C6) and upper thoracic spinal cord injuries.

**Methods:**

Participants of the present study were the patients diagnosed with ASIA A or B. The patients were divided into two groups according to their spinal cord injuries: C6 group and T2-T6 group. The level of the desk was set at 5 cm below the elbow, at the elbow level, and 5 cm above the elbow level. Electromyography was used to measure the duration of typing task EMG(%RVC) of the cervical erector spinae, upper trapezius, anterior deltoid, and wrist extensor. Subjective musculoskeletal discomfort (Borg-RPE) was measured at the end of the experiment.

**Results:**

The two groups showed differences in terms of RPE corresponding to each level of the computer desk (*p* < .05). Postanalysis revealed the C6 group had decreased RPE as the level of computer desk increased, whereas the subjects in the T2-T6 group had decreased RPE values in accordance with the decreasing level of computer desk (*p* < .05). In EMG, both groups had no significant differences (*p* > .05). However, in terms of the interaction between the muscles and the level of computer desk in both groups, the differences in the interactions of the upper trapezius and wrist extensor with each level of the desk were found (*p* < .05).

**Conclusion:**

This study is meaningful in that it confirms computer work posture and preference of spinal cord-injured individuals.

## 1. Introduction

The global incidence of spinal cord injury shows an increasing trend every year. Approximately one-third of patients with spinal cord injuries also have tetraplegia, wherein approximately 50% of the patients have complete injuries [1, 2]. The injuries are mainly due to various accidents, including the increasing incidence of industrial accidents; the increase in the number of patients is also ascribable to advancements in medical science which have resulted in increased cases of the survival of patients with disabilities [3]. Spinal cord injury is typically accompanied by neuroplegia of the motor and sensory nerves as well as various physical disorders [4]. Spinal cord injury is an injury in the central nervous system, which is difficult to completely treat. Rehabilitation of patients with spinal cord injuries necessitates biomedical and complementary approaches simultaneously with sociocultural approaches [5].

The number of working environments involving interfaces with computers has been increasing recently, wherein the majority of employees spend an average of 7 hours a day working at the interfaces to complete their work [6]. Individuals with disabilities are able to work and socialize using computers [7, 8]. Along with the increase in the number of computer users, the number of patients with neck and shoulder pains and problems in the musculoskeletal system, such as carpal tunnel syndrome, has also been increasing [9, 10]. Studies determining the proper ergonomic postures fit for individuals with sedentary work, such as those work involving computers, have been increasing lately [11]. However, most studies that explored proper ergonomic postures required for working with computer interfaces have been carried out in the European and North American environment, thereby the results of such studies may not comply with physical conditions of Asian people. In particular, the results may not be suitable for Asian people relying on wheelchairs [12]. For patients with spinal cord injuries, the risk of musculoskeletal problems resulting from the use of VDTs would be higher than normal people owing to the difficulty in diminishing static load or controlling respective postures wherein respective patients would have different postures with which they feel comfortable [12–14]. Currently, the majority of studies have involved healthy individuals, and the number of studies investigating the proper postures required when using computers or preferences of patients with spinal cord injuries is insufficient.

Therefore, the present study is aimed at identifying the effects of computer desk heights on musculoskeletal discomfort of the neck and upper extremities and EMG (electromyography) activities of patients with spinal cord (C6) and upper thoracic spinal cord injuries (T2-T6).

## 2. Methods

### 2.1. Participants

A total of 12 patients admitted to the Y-rehabilitation Hospital located in Gyeonggi-do province from January 17 to January 26, 2018, were included in the present study. They were divided into the following two groups according to the level of spinal cord injury: C6 group (six patients) and T2-T6 groups (six patients).

The inclusion criteria were as follows: (1) patients diagnosed as having complete injuries of motor function of the types of either ASIA A or B defined by the American Spinal Injury Association (ASIA) without cerebral injuries and complications such as bone fracture or bedsores [15–17]; (2) male sex to eliminate differences in physical measurements attributable to different sexes [18]; (3) right-handed; (4) over 0.8 corrected vision; (5) normal range of articular motion of the upper extremities; and (6) understood the purposes of the present study and provided informed consent to participate in the present study [17].

### 2.2. Experimental Method

**Figure 1:**
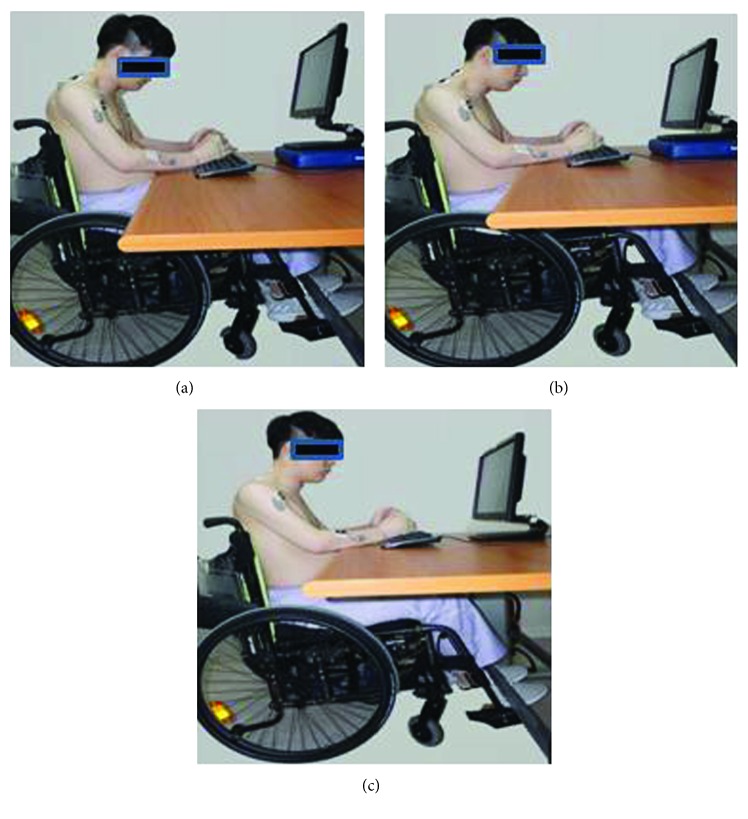
Desk height.

The study protocol was approved by the IRB of Hallym University (no. HIRB-2017-064) and adhered to the ethical principles of the Declaration of Helsinki. The computer used for the study had a 21-inch monitor with adjustable levels, wherein the top of the monitor was set at the patient's eye level. The distance from the monitor to the eyes of the patients was set at 70 cm, rather than the recommended range of 63 to 93 cm, in consideration of the patients' physical characteristics as subjects and their positions were each monitored into account [19]. Keyboards used for the experiment were the standard ones. The keyboards were placed on each desk with “G” and “H” keys placed at the center of each patient and with a keyboard slope angle of 3°, which was set as the default angle [20]. Based on the study on subjective preference and fatigability of spinal cord-injured patients who use wheelchairs, the height of the keyboard was set to 5 cm below the elbow, the height of the elbow, and 5 cm above the elbow [12], and the height adjustable desk were used for the matter of the heights [21]. The participants were allowed to use their wheelchairs for the experiment. Participants were asked to sit at 90°-110° hip joint angle and over 90° knee joint angle [17]. For the C6 group, the “bend-type typing device” was used [7]. The participants were asked to type for two minutes; a metronome was used to eliminate the effects resulting from the participants' different typing speeds. A simple typing task was introduced to minimize the factors affecting EMG activities of the participants, which could lead to psychological reactions of each subject who might be unfamiliar to the working environment and input data [22, 23]. The subjects were asked to use their right hand to enter “J,” “K,” and “L” keys, while their left hand were supposed to type the “A,” “S,” “D,” and “F” keys, simultaneously. The participants were asked to perform the typing task three times with one-minute rest time between the typing tasks at identical keyboard level. The sequence set to complete the typing tasks of each subject was determined randomly. Five minutes of time for resting upon completion of each typing task with the given posture was allowed for each subject. The participants freely placed their arms on their legs during the rest time [17]. Moreover, the participants were instructed not to put their wrists and forearms on the computer desk to eliminate the effects resulted from the support of the lower arms during the given typing task [17] (Figure 1). The subjective musculoskeletal discomforts in the cervical erector spinae, upper trapezius, anterior deltoid, and wrist extensor were measured using the “Rating of Perceived Exertion Scale,” and EMG activities of the neck and upper extremities of the subjects corresponded to the varied positions of the keyboard. The cervical erector spinae and upper trapezius frequently exhibit muscular pains and diseases resulting from an accumulation of myalgia. The muscles used in long durations would cause musculoskeletal problems due to the muscles being activated continuously to maintain the static postures required [24]. In individuals doing repetitive typing tasks using the hands and arms, the musculoskeletal diseases would arise from the arm, shoulder girdle, and wrist [25, 26]. In particular, the anterior deltoid would be affected by the level of the keyboard. Moreover, the continuous repetitive tasks would result in musculoskeletal problems in the wrist extensor [17] Thus, the four muscles, which are likely to have frequent problems in individuals working on computers, were evaluated in this study. Furthermore, the Borg-RPE (Rating of Perceived Exertion) scale was employed for the measurement of musculoskeletal discomforts of the neck and upper extremities. The RPE scale spanned the range from “no pain at all” to “maximal pain” [27]. The scores ranged from 6 points (the minimum) to 20 points (the maximum), with the lower scores indicating less discomfort [28]. The scores were obtained from self-report checklists distributed to each subject upon completion of the experiment.

### 2.3. Data Collection

**Figure 2:**
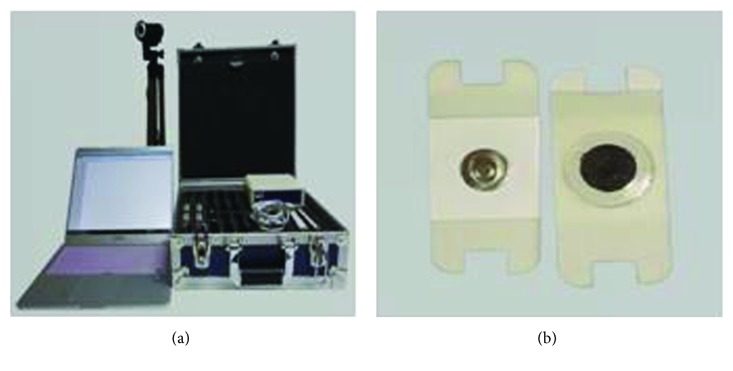
EMG measuring tools.

**Figure 3:**
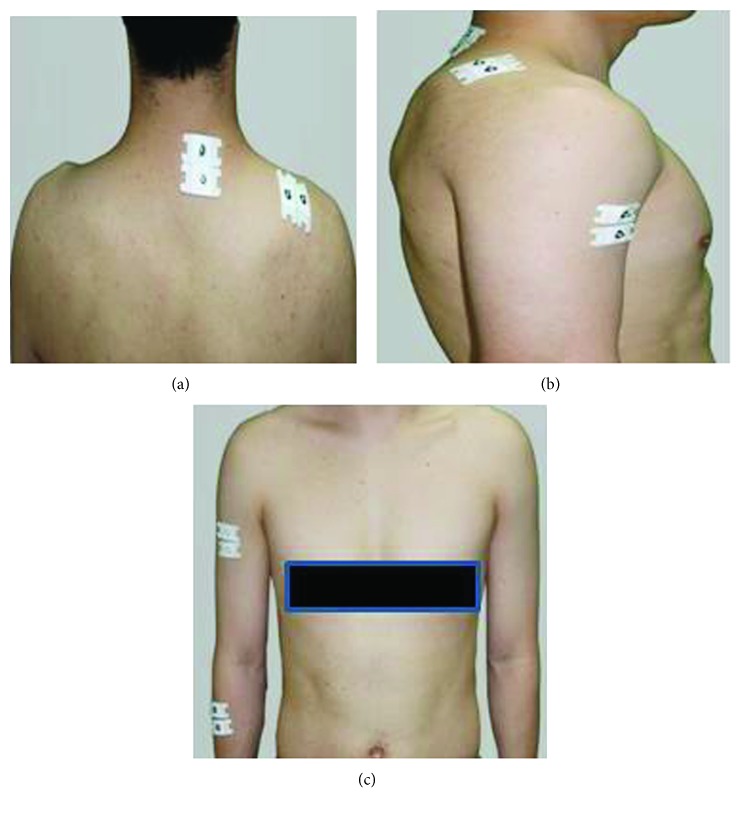
EMG attachment part (posterior, lateral, and anterior view).

During the task, EMG measurement was performed using a wireless EMG system (Wave EMG Infinity Waterproof, Cometa System Inc., Italy) (Figure 2). Adhesive dual electrodes of Ag-AgCl type, which were fixed 2 cm apart, were used as surface electrodes. The “visual-3D” was used for its analysis. The sampling rate was set at 2,000 Hz for electromyogram signals. The frequency range was set at the interval of 30-250 Hz for the band-pass filtering. The measured signals of electromyogram were rectified and then smoothed by employing the “Root Mean Square (RMS)” method [29]. While the participants were performing the typing task, the electrodes were attached to the right cervical erector spinae, upper trapezius, anterior deltoid, and wrist extensor, based on the SENIAM (Surface ElectroMyography for the Noninvasive Assessment of Muscles) instructions [30] (Figure 3). The measurements of the electromyogram signals, collected during the typing task, were standardized according to the muscular contraction of specified muscles, also termed “Reference Voluntary Contraction (RVC)”. These values were then standardized as %RVC [31, 32]. RVC of the neck muscle was measured by asking the participants to wear a 0.3 kg helmet and to maintain their posture with the head erected for 10 seconds. RVC of the shoulder muscle was measured by asking participants to wear a 0.3 kg sandbag on their right wrist. RVC of the upper trapezius was measured with the patient's arm abducted at 90° for 10 seconds, whereas the RVC of the anterior deltoid was measured with the arm bent at 90°. RVC of the wrist extensor was measured by asking the patient to carry a 0.3 kg dumbbell with the wrist maximally extended for 10 seconds [25]. The pads were attached by a single person through the experiment to reduce errors in the measurement of electromyogram. Electromyograms, obtained from varied measurement durations of 100 seconds to 300 seconds, are used for various purposes and tasks reported in previous studies [17, 33]. In the present study, a simple task was used in consideration of patients with spinal cord injuries. The duration was set at 140 seconds, wherein the electromyogram obtained from the duration of 120 seconds which resulted from an exclusion of 10 seconds at both ends of the interval was used for the analyses conducted in the present study [23, 34]. In addition, musculoskeletal discomforts were measured upon completion of all experiments using the self-report checklist and the RPE Scale [28].

### 2.4. Data Analysis

The PASW 22.0 (IBM/SPSS Inc., Chicago, IL) for Windows was used for the statistical analyses. The patients' general characteristics, such as the age, height, weight, sitting eye level, and sitting elbow height, were expressed in mean values and standard deviations, whereas the date of onset of spinal cord injury, ASIA scale, and level of injuries were expressed in percentage. An ANOVA was carried out to analyze the musculoskeletal discomfort corresponding to the different levels of the computer desk to determine the correct level of computer desk corresponding to each level of injury. Values of %RVC for the four muscles of subjects which corresponded to each level of the computer desk were analyzed by conducting a two-way ANOVA for repetitive measurements. The significance level was set at *α* = 0.05. The postanalytic Dunn-Bonferroni procedure was used to perform multiple comparisons of the variables found to be statistically and significantly different.

## 3. Results

### 3.1. General Characteristics of the Patients

The details of the general characteristics of the subjects are summarized in Table 1.

### 3.2. Correlation of the RPE Values and Computer Desk Height

A significant difference in terms of an indicator of physical discomfort, which varied by different levels of the keyboard of the subjects in the two groups, was found (*p* < .05) (Table 2). The RPE value of the patients in the C6 group decreased when the computer desk height was increased (*p* < .05), whereas, in the T2-T6 group, the RPE value decreased when the computer desk height was decreased (*p* < .05). In particular, the RPE values of the T2-T6 group were significantly different in the following computer desk levels: (1) between the 5 cm below the elbow level and elbow level and (2) between the 5 cm above the elbow level and 5 cm below the elbow level (*p* < .05). However, the RPE values in the computer desk level between the level of the elbow and 5 cm above the elbow level had no significant difference (*p* > .05) (Table 3).

#### 3.2.1. Correlation between the EMG of Patients and the Varied Keyboard Positions

The correlations between the EMG activities of the four muscles and the three different keyboard positions were analyzed. No significant differences in EMG activities of the four muscles were found between the different levels of the computer desk (*p* > .05) (Table 4).

#### 3.2.2. Correlation between the Interactions of the Four Muscles and the Various Computer Desk Levels

The correlations between the interactions of the four muscles and the three different computer desk levels were analyzed. Significant differences were found in the interactions of the upper trapezius and wrist extensor (*p* < .05) (Table 5).

## 4. Discussion

In the present study, we investigated the effects of the varied keyboard positions and computer desk heights on musculoskeletal discomforts and EMG activities of patients with spinal injuries who were asked to perform a typing task on a computer, with the goal of identifying the proper keyboard position and computer desk level for these patients to prevent the occurrence of musculoskeletal problems.

Factors that affect the neck and shoulder muscle tension when handling the tasks involving the use of the keyboard include: first, the incline of the thoracic spine and lumbar spine; second, the posture of the cervical vertebrae; third, the posture of the upper arm; fourth, the position of the keyboard and design; and fifth, computer-working skills and break between the work [35]. In particular, for desk height, most biotechnologists recommend that the position of the “home” button should be placed 3 cm above the elbow during elbow joint flexion, but another study suggests 8 cm [36]. In addition, there is a study showing that the damage to the neck and shoulder areas is from higher positions of the keyboard and monitor and that the keyboard should be positioned below the elbow [37]. However, the criteria for these table heights are for the normal person. In a study of patients who use the wheelchair, the study examined the subjective preference and fatigability of users over 5 cm and below 5 cm based on the elbow height and found that spinal cord-injured patients prefer the keyboards that were located at the height of the elbow or below 5 cm [12]. In the case of a normal person, muscle activity was doubled when working on the table that is 5 cm higher than an elbow-high table. Also, [23] when the location of the keyboard is below the elbow, it reduces the risk of getting damage on the neck and shoulder areas [38]. However, an objective study on whether these results appear the same for spinal cord-injured patients using wheelchairs was needed, so the 5 cm above and below presented in the preceding studies were set to the keyboard height based on the elbow height.

The results of the study showed that the significant differences are found between the RPE values and the varied computer desk levels in both patient groups. Our results were similar to the findings of a previous study involving patients with upper thoracic spinal cord injury who were using wheelchairs which sought to identify subjective preference and degree of fatigue when working on computers with various desk heights similar to ours. Their patients preferred the desk height level of 5 cm below the elbow level [12, 28]. The decrease in the RPE values was considered to be attributed to the muscular tension of the neck and shoulder, which increased when the desk level increased, thereby resulting in tensional neck syndrome and discomfort [20, 34]. However, in the patients with complete injuries of C6, whose finger functions and wrist flexion, as well as the trunk and lower extremity functions are lost, reduced balancing capability occurs [7, 39]. In particular, the injury of the spinal cord would decrease the capability of the trunk adjustment significantly compared to the injury of the thoracic spinal cord [16, 40]. Thus, the reduced musculoskeletal discomfort of the patients with spinal cord injuries might be due to the stability of their trunk, which is increased when the computer desk height is increased.

In the International Standards for Neurological Classification of Spinal Cord Injury (ISNCSCI), the seriousness of the injury is described by using ASIA Impairment Scale. The most severe grade AISA A is considered AISA A because the sensory or motor function is not maintained in the spinal cord S4-5 because the sensor is completely lost [41]. In particular, in the case of a motor function, they only existed within the neck muscle and major muscles from the upper extremity deltoid, elbow flexor, and wrist extensor when the cervical spinal nerve 6 is completely damaged. If hydrothorax 2-6 is completely damaged, the muscles in the neck and the abdomen will remain the same, but the tension in the abdominal muscles will decrease [16]. In the case of the spinal cord-injured patients, especially the upper level, they show a noticeable back bend in the sitting posture and, in reaction to this, a straighten neck position [42]. Therefore, this study selected the cervical erector spinae and upper trapezius as an experimental muscle because their muscle activity influenced a lot due to reducing in the trunk's tension that is for maintaining posture during handling task [43, 44]. In addition, 90% of cumulative trauma disorders that comes from the use of VDT are related with upper extremities [45]. In particular, disease from the hand and wrist showed the greatest frequency, followed by the neck, arms and shoulders [46]. The form of standardized keyboard requires hyperextension of the wrist joint, and the work station's location requires lifting the upper arm which leads to tiredness of deltoid [45]. Therefore, the wrist extensor and deltoid that influence a lot by the form of keyboard and the height of desk when handling the task were selected as experimental muscles.

Moreover, the effects of the different keyboard positions on EMG activities of patients with spinal cord injuries during the typing task were explored. No significant differences were found between the varied keyboard positions and EMG activities. The Occupational Safety and Health Administration (OSHA) of the United States of America recommends that the appropriate keyboard position should be from 70° to 135° of elbow flexion. However, these recommended levels are only applicable in healthy individuals; hence, studies investigating the effects of computer desk level of work table on the upper extremities, necks, and shoulders of patients with spinal cord injuries using wheelchairs are warranted [47]. Furthermore, the correlation between the four muscles and the different computer desk levels was analyzed. The significant differences were found in interactions of the two muscles of upper trapezius and wrist extensor of the subjects in both groups with the varied level of the computer desk, which might be attributable to the effects of the level of computer desk over the upper trapezius of which major function was originally supposed to raise the scapula [48]. In addition, the wrist extensor of the patients in the C6 group was found to be affected more by the varied levels of the computer desk than those in the T2-T6 group, which might be due to their difficulty in controlling their forearm because of the complete loss of finger functions, partial loss of wrist joint functions, and weak pronator and supinator [49, 50]. Moreover, as the wrist extensor and anterior deltoid were found to be affected significantly by the varied positions of the keyboard as reported previously, the significant difference in the interaction between the function of wrist extensor and varied level of computer desk could be ascribable thereto [17, 34, 50].

The population of disabled people in Korea was estimated to be approximately 2,510,000 in 2014, wherein the individuals with spinal cord injuries account for 4.9% of the population with limb and body disabilities [51]. Hence, their quality of life has received much attention, which is reflected by several previously conducted studies. Factors affecting the quality of life of these patients include level of economic status, sex life, social support, the feeling of helplessness, depression, the degree of performance in daily living, occupational activities, and mobility, among others [52]. In particular, the availability of the Internet was found significantly associated with the quality of life; the emotional states, physical states, a functional domain, economic life, and self-esteem were also found significantly associated with their quality of life [53]. In terms of the disability resulting from the spinal cord injury, tetraplegia due to injuries in the cervical spinal cord would be more serious than paraplegia due to the thoracic spinal cord injury [54], thereby the tetraplegic patients need auxiliary tools when working on computers and environmental provisions [17]. However, only a few studies investigated on the working conditions, relating to computer use, of patients with spinal cord injuries. Considering the current situations, wherein engineering approaches employing computers to assist patients with cervical spinal cord injuries are increasing, the present study would be significant because it provides objective experiments employing EMG activities and indicators of subjective musculoskeletal discomfort on the varied desk levels.

The study had several limitations. First, the study has a small sample size. Second, the representativeness of the included patients was difficult to secure despite the homogeneity of the patient's general characteristics to avoid selection bias in the study participants. Third, the typing task was a simplified short-run task, which was remarkably different from the actual work performed in computers. Thus, future studies with a large sample size and varied computer task to examine EMG activities are warranted.

## 5. Conclusions

The RPE values, which represent the subjective measurement of musculoskeletal problems of the upper extremities of the patients in the C6 group, decreased at the computer desk level of 5 cm above the elbow level, whereas the patients in the T2-T6 group had decreased RPE values at the computer desk level of 5 cm below the elbow. Moreover, the subjects in both groups commonly exhibited no significant differences in EMG activities corresponding to the different levels of the keyboard. However, the upper trapezius and wrist extensor, among the four muscles, were found to be associated with the varied computer desk levels in both groups.

The appropriate computer working conditions should be customized according to the need of the individual with a disability.

## Figures and Tables

**(a):** 

Classification	C6 group (*n* = 6) *M* ± SD	*p*	T2-T6 group (*n* = 6) *M* ± SD	*p*
Age (years)	33.8 ± 15.0	.041	53.3 ± 13.0	.097
Height (cm)	174.7 ± 7.0	.918	172.0 ± 5.9	.497
Weight (kg)	75.1 ± 9.3	.708	70.8 ± 11.3	.567
Eye height in sitting position (cm)	127.8 ± 4.76	.305	122.9 ± 4.5	.166
Elbow height in sitting position (cm)	74.8 ± 3.8	.196	73.3 ± 2.9	.111

**(b):** 

Classification	C6 group (*n* = 6)		T2-T6 group (*n* = 6)	
	Number (*n*)	Percent (%)	Number (*n*)	Percent (%)
Onset (year)				
Less than 1	1	16.7	4	66.7
1 to 2	4	66.7	2	33.3
2 or over	1	16.7	0	0
ASIA				
A	5	83.3	3	50
B	1	16.7	3	50
Level of injury	C6 (*n* = 6)	C6 (100)	T2 (*n* = 3), T6 (*n* = 3)	T2 (50), 6 (50)

^∗^ *p* < .05 and values are *n* (%) or mean ± standard deviation.

**Table 2:** Correlation between RPE and computer desk height (*n* = 12).

	Desk height	Body part discomfort rating (RPE)	*p*	*F*
C6	5 cm below	15.33 ± 1.50	^∗^.000	17.245
	Same as	13.00 ± 1.26		
	5 cm above	10.33 ± 1.63		

T2-T6	5 cm below	10.33 ± 1.03	^∗^.001	12.297
	Same as	12.33 ± 1.03		
	5 cm above	14.00 ± 1.67		

^∗^ *p* < 0.05.

**Table 3:** Posthoc analysis of the musculoskeletal discomfort and desk height (*n* = 12).

	Group	Desk height		MD	SE	*p*
Body part discomfort rating (RPE)	C6	5 cm below	Same as	2.333	.852	^∗^.046
		5 cm below	5 cm above	5.000	.852	^∗^.000
		Same as	5 cm above	2.666	.852	^∗^.021
	T2-T6	5 cm below	Same as	−2.000	.740	^∗^.049
		5 cm below	5 cm above	−3.666	.740	^∗^.001
		Same as	5 cm above	−1.666	.740	^∗^.119

^∗^ *p* < 0.05; MD: mean difference; SE: standard error.

**Table 4:** Comparison of the EMG of the 2 groups of subjects corresponded to varied positions of keyboard (*n* = 12).

	C6 group Keyboard position (*n* = 6)					T2-T6 group Keyboard position (*n* = 6)				
	Elbow flexion			*p*	X2/*F*	Elbow flexion			*p*	X2/*F*
	5 cm elbow	Same as	5 cm above			5 cm below	Same as	5 cm above		
CES	277.27 ± 125.27	301.79 ± 146.10	303.67 ± 152.70	.810	.422	187.05 ± 68.29	420.94 ± 555.51	231.23 ± 84.19	.440	.868
UT	11.38 ± 10.78	16.32 ± 16.55	17.31 ± 21.99	.458	.796	22.71 ± 10.03	32.36 ± 18.16	41.23 ± 21.11	.135	4.012
AD	19.43 ± 9.11	19.42 ± 7.62	19.42 ± 7.78	.910	.188	21.20 ± 9.05	21.39 ± 7.86	24.19 ± 11.57	.692	.737
WE	53.32 ± 19.83	54.75 ± 22.15	59.43 ± 20.81	.611	.986	33.08 ± 15.08	31.80 ± 13.99	34.56 ± 12.27	.849	.327

^∗^ *p* < .05; CES: cervical erector spinae; UT: upper trapezius; AD: anterior deltoid; WE: wrist extensor.

**Table 5:** Comparison of interactions of the four muscles of subjects in the two groups with the varied levels of the computer desk (*n* = 12).

	Desk	CES interaction (group × desk)			UT interaction (group × desk)			AD interaction (group × desk)			WE interaction (group × desk)		
		*M* ± SD	*p*	*F*	*M* ± SD	*p*	*F*	*M* ± SD	*p*	*F*	*M* ± SD	*p*	*F*
C6	5 cm below	277.28 ± 125.27	.629	.585	11.38 ± 10.78	^∗^.013	4.196	19.43 ± 9.11	.758	.394	53.32 ± 19.83	^∗^.006	4.989
	Same as	301.79 ± 146.10			16.33 ± 16.55			19.42 ± 7.62			54.76 ± 22.15		
	5 cm above	303.67 ± 152.70			17.31 ± 21.99			19.42 ± 7.78			59.44 ± 23.75		
T2-T6	5 cm below	187.05 ± 68.29			22.71 ± 10.03			21.20 ± 9.05			33.08 ± 15.08		
	Same as	420.94 ± 555.51			32.37 ± 18.16			21.39 ± 7.86			31.81 ± 13.99		
	5 cm above	231.24 ± 84.19			41.23 ± 21.11			24.19 ± 11.57			34.57 ± 12.27		

^∗^ *p* < .05, mean ± standard deviation.

## Data Availability

The demographics and clinical data collected to support the findings of this study are restricted by the Ethics Committee of the Province of Hallym University (Republic of Korea) in order to protect patient privacy. Data are available from Bo-Ra Kang, Yonseimadu Hospital 123, Gangseok-ro, Ilsandong-gu, Goyang-si, Gyeonggi-do, Republic of Korea (violet781@nate.com), for researchers who meet the criteria for access to confidential data.
